# The impact of gender, level of amputation and diabetes on prosthetic fit rates following major lower extremity amputation

**DOI:** 10.1177/0309364616628341

**Published:** 2016-02-05

**Authors:** Fiona Davie-Smith, Lorna Paul, Natalie Nicholls, Wesley P Stuart, Brian Kennon

**Affiliations:** 1 University of Glasgow, Glasgow, UK; 2 MRC, Glasgow, UK; 3 NHS GGC, Diabetes Centre, Southern General Hospital, Glasgow, UK

**Keywords:** Diabetes, prosthetics, rehabilitation

## Abstract

**Background:**

Diabetes mellitus is a leading cause of major lower extremity amputation.

**Objective:**

To examine the influence of gender, level of amputation and diabetes mellitus status on being fit with a prosthetic limb following lower extremity amputation for peripheral arterial disease.

**Study design:**

Retrospective analysis of the Scottish Physiotherapy Amputee Research Group dataset.

**Results:**

Within the cohort with peripheral arterial disease (*n* = 1735), 64% were men (*n* = 1112) and 48% (*n* = 834) had diabetes mellitus. Those with diabetes mellitus were younger than those without: mean 67.5 and 71.1 years, respectively (*p* < 0.001). Trans-tibial amputation:trans-femoral amputation ratio was 2.33 in those with diabetes mellitus, and 0.93 in those without. A total of 41% of those with diabetes mellitus were successfully fit with a prosthetic limb compared to 38% of those without diabetes mellitus. Male gender positively predicted fitting with a prosthetic limb at both trans-tibial amputation (*p* = 0.001) and trans-femoral amputation (*p* = 0.001) levels. Bilateral amputations and increasing age were negative predictors of fitting with a prosthetic limb (*p* < 0.001). Diabetes mellitus negatively predicted fitting with a prosthetic limb at trans-femoral amputation level (*p* < 0.001). Mortality was 17% for the cohort, 22% when the amputation was at trans-femoral amputation level.

**Conclusion:**

Of those with lower extremity amputation as a result of peripheral arterial disease, those with diabetes mellitus were younger, and more had trans-tibial amputation. Although both age and amputation level are good predictors of fitting with a prosthetic limb, successful limb fit rates were no better than those without diabetes mellitus.

**Clinical relevance:**

This is of clinical relevance to those who are involved in the decision-making process of prosthetic fitting following major amputation for dysvascular and diabetes aetiologies.

## Background

Major lower extremity amputation is associated with significant morbidity and mortality. Worldwide, diabetes mellitus (DM) is the foremost cause of major lower extremity amputations,^1^ and in the United Kingdom, DM accounts for 5–6000 major amputations each year.^2^ Despite evidence that multi-disciplinary DM foot services reduce amputation rates,^3^ a significant proportion of people with will undergo a major amputation. A recent study from Scotland found the major amputation rate to be 1.39 per 1000 persons with DM.^3^ Given the socioeconomic burden, significant mortality and adverse impact on quality of life associated with major amputation,^4–6^ optimising function post-operatively is of vital importance.

Although DM has not been shown to influence survival rates in the immediate post-amputation period,^1,7^ there is evidence that in the longer term the presence of DM reduces survival by almost 50% with a mean life expectancy of 27.2 months compared to 46.7 months in people with and without DM respectively.^8^ DM has also been shown to double the likelihood of requiring a second or contralateral amputation.^1,9^

Another important factor associated with life expectancy is the level of amputation.^10–12^ Subramaniam et al.^13^ reported the 30-day mortality following trans-femoral amputation (TFA) (18%) to be four times higher than that of trans-tibial amputation (TTA) (4%). Given the adverse impact that DM has on life expectancy following major amputation, it is important to optimise post-operative function both in terms of quality of life and cardiovascular fitness. Davis et al.^14^ suggested that fitting with a prosthesis impacts significantly upon ambulation and subsequently improves quality of life.

Despite the beneficial effect of being fit with a prosthetic limb, a retrospective study of major lower extremity amputations (LEAs) in a cohort of people with peripheral arterial disease (PAD) (almost half of whom had DM) demonstrated that only 45% of people ever achieve such an outcome.^15^ Factors that affect the process of being fit with a prosthetic limb include pre-operative mobility, amputation level, age and the presence of coronary artery disease.^15–17^ There is limited evidence examining the impact of DM on the chances of being successfully fit with a prosthetic limb post major amputation.

The aim of this study was to examine the impact of DM, gender and the level of amputation on the rates of being fit with a prosthetic limb following non-traumatic major amputation in Scotland from 2007 to 2009, utilising data from the Scottish Physiotherapy Amputee Research Group (SPARG). Limb fitting rates were recorded up to discharge from rehabilitation services.

## Materials and methods

This retrospective cohort study examined persons who underwent a non-traumatic major amputation, at TF and TT levels, in Scotland from 1 January 2007 to 31 December 2009. Ethical approval for the study was given by the College of Medical, Veterinary and Life Sciences (MVLS), Ethics Committee, University of Glasgow. The reason for amputation was PAD in the presence or absence of DM. Amputations due to tumour, trauma, congenital abnormality, orthopaedic complications or intra-venous drug use were excluded. Also excluded were amputations at the level of hip disarticulation, trans-pelvic, through knee and ankle disarticulation, as these were in small numbers and have specific requirements for rehabilitation and prosthetic manufacture.

Major LEA was defined as a complete loss of the limb in the transverse anatomical plane proximal to the ankle.^18^ The final recorded level of amputation was taken as the most proximal, capturing those who underwent revisions to a higher level. If a person underwent bilateral amputation within the 3-year period, this was also recorded. The presence of DM at the time of major amputation was confirmed from the medical notes but not categorised further.

Data were extracted from the SPARG dataset. SPARG is a network of physiotherapists with clinical responsibility for people with a major amputation in all the health boards of Scotland. Anonymised data have been prospectively collected from all people undergoing major amputation in Scotland since 1991. The dataset consists of demographics, rehabilitation milestones and limb fitting outcomes.^19^ Data were collected from the time of amputation until discharge from the rehabilitation service. This included out-patient treatment.

The following variables were extracted from the SPARG database: gender, age, DM status, Functional Co-Morbidity Index (FCI) (an 18-point tool that measures presence of co-morbidities)^20^ at time of amputation, date of amputation, primary indication for amputation (underlying pathology), the level and side of amputation, number of previous amputations, length of in-patient and out-patient treatment, discharge destination, dates and final outcome at the end of the rehabilitation process.

Final outcomes were categorised as follows: limb fit (LF) (discharged from rehabilitation using a prosthesis), non-limb fit (NLF) (did not start the limb fitting process), abandoned (commenced the prosthetic process and then stopped prior to discharge from rehabilitation) or died (death during the rehabilitation process).

Descriptive statistics were undertaken for the total dataset, and then specifically for those with and without DM. Chi-squared tests of association were used for comparison between populations; a binary logistic model was used to determine the likelihood of being fit with a prosthetic limb following a TT or TFA. All analysis was performed using Minitab v15 and R software packages, with a significance level of 5%.

## Results

There were 2145 major amputations performed in Scotland between 1 January 2007 and 31 December 2009. In 331 cases, the major amputation was due to tumour, trauma, congenital abnormalities, orthopaedic complications or intra-venous drug use and these were excluded from further analysis. Amputations at hip disarticulation level (*n* = 17), trans-pelvic (*n* = 2) and ankle disarticulation (*n* = 4) were also excluded. Forty-four people underwent knee-disarticulation amputations over the 3 years, and of these, 12 people had DM and 26 did not. This group was also excluded from further analysis because they represented a small portion of the cohort (3%) and had quite specific characteristics and requirements for both rehabilitation and prosthetic manufacture. A total of 1747 people underwent amputations at TT and TF levels. In 12 cases (<1%), data were incomplete leaving 1735 cases for analysis.

The cohort consisted of 64% men (*n* = 1112) and 36% women (*n* = 623). Of the total cohort, 48% (*n* = 834) had DM, of which 70% (*n* = 582) were men and 30% (*n* = 252) women. This compared to 59% (*n* = 530) men and 41% (*n* = 371) women in those without DM. The mean age at amputation was 69.7 years (standard deviation (SD) = 12.3). Those with DM were significantly younger (mean = 67.5 years) than those without DM (mean = 71.1 years) (*p* < 0.001); this was true for both men and women (both *p* < 0.001) (Table 1).

**Table 1.** Total cohort gender, diabetes status and age.

	Non-diabetes, *n* (%)	Diabetes, *n* (%)	*p*-value
Gender			
Total	901 (52)	834 (48)	
Men	530 (59)	582 (70)	< 0.001
Women	371 (41)	252 (30)	< 0.001
M/W ratio	1.43	2.31	
Level			
Bilateral	150 (17)	214 (26)	< 0.001
TTA	362 (40)	434 (52)	< 0.001
TFA	389 (43)	186 (22)	< 0.001
TTA/TFA ratio	0.93	2.33	
Mean age (years)	71.1	67.5	< 0.001
Men, mean age (SD)	70.6 (11)	67.4 (11)	< 0.001
Women, mean age (SD)	73.6 (11)	68.7 (13)	< 0.001

M/W: men/women; TTA: trans-tibial amputation; TFA: trans-femoral amputation; SD: standard deviation.

In terms of co-morbidities, FCI scores were generally low (mean = 3.2, SD = 1.9). Scores were similar between men (mean = 3.3) and women (mean = 3.4) and between those with and without DM (mean = 3.8 and 2.9), respectively.

In the overall cohort, 79% (*n* = 1371) had a unilateral amputation, of these 58% (*n* = 796) were TTA and 42% (*n* = 575) were TFA. There was a significantly greater proportion of people with a TTA in the cohort with DM (*p* < 0.001) (Table 1). The people with DM were therefore younger and more likely to have TTAs.

Overall, 38% (*n* = 659) of the cohort were fit with a prosthetic limb. This did not include the group that started but abandoned the process (3%). Overall, 72% (*n* = 477) of those who underwent TTA were fit with a prosthetic limb compared to 16% who underwent TFA (*n* = 94). Twenty-four percent (*n* = 88) of people with bilateral amputations were fit with prosthetic limbs, which is a significantly lower proportion than those with a unilateral amputation (42%, *n* = 571) (*p* < 0.001). In relation to bilateral amputation, the level also impacted on being fit with a prosthetic limb as those with bilateral TFAs were significantly less likely to be fit with prosthetic limbs compared to those with bilateral TTAs (*p* < 0.001) (Table 2).

**Table 2.** Final limb fit outcomes of people with bilateral amputations.

Bilateral level combination	Limb fit, *n* (%)	Non-limb fit, *n* (%)	Abandoned, *n* (%)	Died^a^, *n* (%)
Bilateral TTA (*n* = 196)	87 (44)	74 (38)	8 (4)	27 (14)
TTA + TFA (*n* = 14)	1 (7)	6 (43)	3 (21)	4 (29)
Bilateral TFA (*n* = 154)	0 (0)	119 (77)	0 (0)	35 (23)
Total (*n* = 364)	88 (24)	199 (55)	11 (3)	66 (18)

TTA: trans-tibial amputation; TFA: trans-femoral amputation.

a Died within the rehabilitation period.

In terms of gender, for both TTA and TFA, significantly more men than women were fit with a prosthetic limb (TTA: men = 341 (63%), women = 136 (53%); TFA: men = 65 (21%), women = 29 (11%) (*p* = 0.001)). Gender had no impact on those who abandoned the prosthetic fitting process (Table 3).

**Table 3.** Influence of gender on limb fit depending on amputation level.

		Total	Limb fit, *n* (%)	Non-limb fit, *n* (%)	Abandoned, *n* (%)	Died^a^, *n* (%)
TTA	Total	796	477 (60)	200 (25)	22 (3)	97 (12)
	Male	538	341 (63)	119 (22)	15 (3)	63 (12)
	Female	258	136 (53)	81 (31)	7 (3)	34 (13)
TFA	Total	575	94 (16)	342 (60)	11 (2)	128 (22)
	Male	304	65 (21)	176 (58)	6 (2)	57 (19)
	Female	271	29 (11)	166 (61)	5 (2)	71 (26)
Bilateral	Total	364	88 (24)	199 (55)	11 (3)	66 (18)
	Male	256	69 (27)	133 (52)	8 (3)	46 (18)
	Female	108	19 (17)	66 (61)	3 (3)	20 (19)

TTA: trans-tibial amputation; TFA: trans-femoral amputation.

a Died within the rehabilitation period.

The mortality rate within the rehabilitation period was 17% (*n* = 291); 16% for those with DM and 17% for those without DM. This rate was higher following bilateral amputation (18.1%) and unilateral TFA (22%) compared to unilateral TTA (12%) (Table 3). Thirty-day mortality was 7% (*n* = 114), of which 22% (*n* = 25) had bilateral amputations and 78% (*n* = 89) had unilateral amputations. Furthermore, 43% had DM compared to 57% without DM. The 30-day mortality was 50% in those with bilateral amputation who had one TTA and one TFA and, of these, 75% had DM. In those with bilateral TFAs, 51% died within 30 days and 50% of those had DM. In comparison, 19% of those with bilateral TTAs died within 30 days and all had DM.

Within the cohort, 48% had DM (*n* = 834), and of those 41% (*n* = 346) were fit with a prosthetic limb. With respect to those who underwent TTA, similar proportions of those with and without a diagnosis of DM were fit with a prosthetic limb (44% and 56%, respectively; *p* = 0.399). By comparison, only 16% of people who underwent a TFA proceeded to fit with a prosthetic limb. Of these, significantly fewer people with DM (*n* = 14, 8%) were fit with a prosthetic limb compared to those without DM (*n* = 80, 21%) (*p* < 0.001).

In the group with DM, bilateral amputations were significantly more frequent (*n* = 214) compared to those without DM (*n* = 150) (*p* < 0.001). Additionally, there were more bilateral TTAs compared to bilateral TFAs within the group with DM but this difference was not significant (*p* = 0.070). For those with DM, significantly more men underwent bilateral amputations than women (*p* = 0.009). There was no significant difference in mortality between those with and without DM (*p* = 0.535) (Table 4).

**Table 4.** Influence of diabetes status on limb fitting depending on amputation level.

		Total	Limb fit, *n* (%)	Non-limb fit, *n* (%)	Abandoned, *n* (%)	Died^a^, *n* (%)
TTA	Total	796	477 (60)	200 (25)	22 (3)	97 (12)
	Non-DM	362	209 (58)	97 (27)	11 (3)	45 (12)
	DM	434	268 (62)	103 (24)	11 (3)	52 (12)
TFA	Total	575	94 (16)	342 (60)	11 (2)	128 (22)
	Non-DM	389	80 (21)	219 (56)	8 (2)	82 (21)
	DM	186	14 (8)	123 (66)	3 (2)	46 (25)
Bilateral	Total	364	88 (24)	199 (55)	11 (3)	66 (18)
	Non-DM	150	24 (16)	92 (61)	6 (4)	28 (19)
	DM	214	64 (30)	107 (50)	5 (2)	38 (18)

TTA: trans-tibial amputation; DM: diabetes mellitus; TFA: trans-femoral amputation.

a Died within the rehabilitation period.

A binary logistic regression model was used to predict which characteristics correlated with the likelihood of being fit with a prosthetic limb following TTA and TFA. Those who had died were excluded, and the outcomes were dichotomised to those who were and those who were not fit with a prosthetic limb (those who abandoned fitting with a prosthesis were included in the latter category for this analysis). The small number of cases who underwent a TTA on one side and a TFA on the other (*n* = 10) were excluded from the analysis.

Men were significantly more likely to fit with a prosthesis than women following both TTA (odds ratio (OR) = 1.71, 95% confidence interval (CI) = 1.24–2.36, *p* < 0.001) and TFA (OR = 2.17, 95% CI = 1.36–3.47, *p* = 0.001). The presence of DM did not predict who were fit with a prosthesis in those who underwent a TTA (OR = 1.14, 95% CI = 0.84–1.56, *p* = 0.399), but for those who underwent a TFA having DM negatively influenced the probability of being fit with a prosthetic limb (OR = 0.35, 95% CI = 0.20–0.61, *p* < 0.001). Increasing age was also a negative predictor of prosthetic fitting after both TTA and TFA; for each year increase in age, there was a 3% reduction in the likelihood of fitting with a prosthesis ((TTA: OR = 0.97, 95% CI = 0.96–0.98, *p* < 0.001) and (TFA: OR = 0.97, 95% CI = 0.95–0.97, *p* *<* 0.001)).

The mean length of rehabilitation was 126 days (range = 84–184 days) following major amputation (men: 127 days, SD = 109; women: 125 days, SD = 118) with no significant differences between those with and those without DM (*p* = 0.462). Similarly, the level of amputation did not seem to influence the duration of rehabilitation (*p* = 0.547). Those who were fit with a prosthetic limb had, on average, 100 more days of rehabilitation than those who were not fit with a prosthesis (*p* < 0.001). For those who were fit with a prosthetic limb, women had a significantly longer rehabilitation period (136.7 days) than men (116 days) by an average of 3 weeks (*p* < 0.010). Similar proportions of people with DM were discharged to their home (80%) compared to those without DM (81%) (*p* = 0.248).

## Discussion

We believe this is the first study to report national prospectively collected data on those who were fit with a prosthetic limb following non-traumatic, major LEA at the TT and TF levels. Within this study, 48% had DM, which is similar to rates reported elsewhere^4,10,15,21^ and specifically in Italy where 49% of those undergoing major LEA had DM^22^ and lower than Spain where 57% of those undergoing major LEA had DM.^23^

Those with DM were more likely to undergo TTA (52%) compared to those without DM (40%).^24^ This is probably due to diabetes-related distal arterial disease below the knee with relative sparing of the proximal vessels.^25^ Although the rates of successful fitting with a prosthetic limb were no different between those with and without DM, our data demonstrate that people with DM who have a major amputation are younger and more likely to have a TTA compared to those without DM. Both factors are regarded as favourable predictors of being successfully fit with a prosthetic limb.^15–17,26,27^

Gender is known to influence major amputation rates and, as in previous studies,^7,10,15,28,29^ we found that major amputation was significantly more common in men. This effect of gender was seen following both TTA and TFA. The gender difference was more pronounced in the group with DM, with men:women ratio of 2.31 compared to 1.43 in those without. The higher amputation rates in men may be due to more severe PAD in men than in women.^23^ Furthermore, higher smoking rates in men may be a contributing factor.^30^ Others have suggested that the presence of oestrogen, which improves wound healing, may explain the relatively lower rates of major amputations in women.^30^

We have also shown that gender influences the chances of successful fitting with a prosthetic limb with more men likely to be fit with a prosthesis than women. Women are more commonly affected by specific co-morbidities such as coronary heart disease and stroke^31,32^ which may explain the lower LF rates in women observed in this study. Although the FCI scores were similar in men and women, we did not investigate the relationship between any specific co-morbidity and limb fitting rates. The reasons for the gender difference in amputations and LF rates merit further investigation.

The overall percentage of those being fitted with a prosthetic limb was 38%. This is lower than previous studies which demonstrated limb fitting figures of 45%–55%.^15,16^ This discrepancy is partly explained by the fact that our study analysed both unilateral and bilateral TTA and TFA levels, whereas previous studies have generally focussed on unilateral amputations.^24,27^ In this study, 42% of those with a unilateral amputation were fit with a prosthesis.

As demonstrated previously, the level of amputation significantly influenced the likelihood of being fit with a prosthesis.^15,16,24,26^ People undergoing a TTA are more likely to be fit with a prosthetic limb compared to those with a TFA. This may be related to the reduced energy expenditure required to walk with a TT prosthesis^26^ or may be due to other factors such as pre-amputation mobility and cognitive status.^15–17^ A TF prosthesis can be less comfortable to wear but also requires a higher level of cognitive function to benefit from the varying knee components.^33^ Only 16% of people were fit with a prosthesis following TFA. A novel finding from the study was the significantly negative impact that DM had on successfully being fitted with a limb specifically following TFA (OR = 0.35). It is likely that complications associated with DM such as obesity, cardiovascular disease, contralateral foot problems, poor balance, poor wound healing and renal insufficiency may explain this finding.

Receiving bilateral amputations has been shown to significantly reduce the likelihood of being fit with a prosthesis and subsequently achieving mobility.^16^ From our study, if a person has bilateral amputations with one at TF level, then they are far less likely to proceed to being fit with a prosthetic limb compared to those with bilateral TTA (Table 5). Also within our study, only 3% of people abandoned the prosthetic fitting process, suggesting that the decision to proceed to being fit with a prosthesis is generally appropriate.

**Table 5.** Predictors of limb fit post major amputation.

Characteristic	Odds ratio (95% CI)	*p*-value
TTA		
Men	1.71 (1.24–2.36)	< 0.001
Diabetes	1.14 (0.84–1.56)	0.399
Bilateral	0.33 (0.23–0.47)	< 0.001
Age	0.97 (0.96–0.98)	< 0.001
TFA		
Men	2.17 (1.36–3.47)	< 0.001
Diabetes	0.35 (0.20–0.61)	< 0.001
Bilateral	0.06 (0.02–0.20)	< 0.001
Age	0.97 (0.95–0.97)	< 0.001

CI: confidence interval; TTA: trans-tibial amputation; TFA: trans-femoral amputation.

The mean age of the entire cohort at time of amputation was 69.7 years. Those with DM were on average 4 years younger, with no significant differences in ages between genders. Similar age differences have been shown in populations of people with amputations in other studies in the United Kingdom,^6^ Greece^1^ and Denmark.^34^ As previously stated, despite persons with DM being younger and with more TTAs, they were no more likely to be successfully fit with a prosthetic limb.

We found that those who were fit with a prosthetic limb spent a longer time in rehabilitation. This is to be expected as prosthetic fitting in itself warrants more physiotherapy input.^1,16^ The finding that women spent longer in rehabilitation whether they were fit with a prosthetic limb or not was novel and is another area that warrants further investigation.

The overall mortality rate within the rehabilitation period was 17%. Previous studies tend to quote 30-day mortality which was 7% in this study, slightly lower than reported in other series at 10%.^11,15,35^ In agreement with other studies, gender and DM status did not influence mortality; however, those with unilateral or bilateral TFAs had higher mortality rates.^15,34^ Subramaniam et al.^13^ report a TFA as a high cardiac risk surgery compared to a TTA as intermediate risk, with mortality rates of 17.5% and 4.2%, respectively.

One of the main strengths of our study is the large and comprehensive nature of the cohort, and by assessing national data we reduce the impact of regional bias and variation in practice within individual units.^29^ However, the use of secondary data analysis has a number of limitations. No cause and effect relationship can be determined between the variables. In addition, we were unable to assess other factors that may influence the ability to successfully fit with a prosthetic limb, such as pre-amputation limb salvaging surgery and cognition^15–17^ as these data are not recorded within the SPARG database. In terms of other limitations, the study only included data from those with TTA and TFA levels of amputation, and included data were recorded only up to the end of the rehabilitation period, on average 4 months from amputation, thus considering only short-term outcomes. Being fit with a prosthetic limb was the only outcome assessed in this study, and future research should consider function, mobility and, importantly, quality of life as specific outcomes.

In conclusion, our large national study of limb fitting rates following major amputation has reinforced previous findings and highlighted more novel aspects related to gender and DM status. Further longitudinal, prospective research is required to investigate factors that influence post-amputation limb fitting rates in persons with DM and in particular women as this will dictate the requirements of clinical services in optimising both the short- and long-term future for this population.

## References

[1] Papazafiropoulou A Tentolouris N Soldatos R-P Mortality in diabetic and nondiabetic patients after amputations performed from 1996 to 2005 in a tertiary hospital population: a 3-year follow-up study. J Diabetes Complications 2009; 23(1): 7–11. 18413176 10.1016/j.jdiacomp.2007.11.008

[2] The Amputee Statistical Database for the United Kingdom 2006/07 Information Services Division, NHS Scotland on behalf of National Amputee Statistical Database (NASDAB) 2009.

[3] Kennon B Leese GP Cochrane L Reduced incidence of lower-extremity amputations in people with diabetes in Scotland: a nationwide study. Diabetes Care 2012; 35(12): 2588–2590. 23011727 10.2337/dc12-0511 PMC3507601

[4] Holman N Young RJ Jeffcoate WJ. Variation in the recorded incidence of amputation of the lower limb in England. Diabetologia 2012; 55(7): 1919–1925. 22398645 10.1007/s00125-012-2468-6

[5] Peters EJ Childs MR Wunderlich RP Functional status of persons with diabetes-related lower-extremity amputations. Diabetes Care 2001; 24(10): 1799–1804. 11574445 10.2337/diacare.24.10.1799

[6] Ebskov LB. Dysvascular amputations and long-term survival in a 20-year follow-up study. Int J Rehabil Res 2006; 29(4): 325–328. 17106350 10.1097/MRR.0b013e328010c78a

[7] Izumi Y Satterfield K Lee S Mortality of first-time amputees in diabetics: a 10-year observation. Diabetes Res Clin Pract 2009; 83(1): 126–131. 19097667 10.1016/j.diabres.2008.09.005

[8] Schofield CJ Libby G Brennan GM Mortality and hospitalization in patients after amputation: a comparison between patients with and without diabetes. Diabetes care 2006; 29(10): 2252–2256. 17003302 10.2337/dc06-0926

[9] Uzzaman MM Jukaku S Kambal A Assessing the long-term outcomes of minor lower limb amputations: a 5-year study. Angiology 2011; 62(5): 365–371. 21421619 10.1177/0003319710395558

[10] Carmona GA Hoffmeyer P Herrmann F Major lower limb amputations in the elderly observed over ten years: the role of diabetes and peripheral arterial disease. Diabetes Metab 2005; 31(5): 449–454. 16357788 10.1016/s1262-3636(07)70215-x

[11] Aulivola B Hile CN Hamdan AD Major lower extremity amputation: outcome of a modern series. Arch Surg 2004; 139(4): 395–399. 15078707 10.1001/archsurg.139.4.395

[12] Kulkarni J Pande S Morris J. Survival rates in dysvascular lower limb amputees. Int J Surg 2006; 4(4): 217–221. 17462354 10.1016/j.ijsu.2006.06.027

[13] Subramaniam B Pomposelli F Talmor D Perioperative and long-term morbidity and mortality after above-knee and below-knee amputations in diabetics and nondiabetics. Anesth Analg 2005; 100(5): 1241–1247. 15845661 10.1213/01.ANE.0000147705.94738.31

[14] Davis BL Kuznicki J Praveen SS Lower-extremity amputations in patients with diabetes: pre- and post-surgical decisions related to successful rehabilitation. Diabetes Metab Res Rev 2004; 20(Suppl. 1): S45–S50. 15150814 10.1002/dmrr.445

[15] Lim TS Finlayson A Thorpe JM Outcomes of a contemporary amputation series. ANZ J Surg 2006; 76(5): 300–305. 16768686 10.1111/j.1445-2197.2006.03715.x

[16] Taylor SM Kalbaugh CA Blackhurst DW Preoperative clinical factors predict postoperative functional outcomes after major lower limb amputation: an analysis of 553 consecutive patients. J Vasc Surg 2005; 42(2): 227–235. 16102618 10.1016/j.jvs.2005.04.015

[17] Sansam K Neumann V O’Connor R Predicting walking ability following lower limb amputation: a systematic review of the literature. J Rehabil Med 2009; 41(8): 593–603. 19565152 10.2340/16501977-0393

[18] Krishnan S Nash F Baker N Reduction in diabetic amputations over 11 years in a defined U.K. population: benefits of multidisciplinary team work and continuous prospective audit. Diabetes care 2008; 31(1): 99–101. 17934144 10.2337/dc07-1178

[19] Condie E Jones D Treweek S; Group SPAR. A one-year national survey of patients having a lower limb amputation. Physiotherapy 1996; 82(1): 14–20.

[20] Groll DL To T Bombardier C The development of a comorbidity index with physical function as the outcome. J Clin Epidemiol 2005; 58(6): 595–602. 15878473 10.1016/j.jclinepi.2004.10.018

[21] Johannesson A Larsson G-U Ramstrand N Outcomes of a standardized surgical and rehabilitation program in transtibial amputation for peripheral vascular disease: a prospective cohort study. Am J Phys Med Rehabil 2010; 89(4): 293–303. 20134308 10.1097/PHM.0b013e3181cf1bee

[22] Lombardo FL Maggini M de Bellis A Lower extremity amputations in persons with and without diabetes in Italy: 2001–2010. PLoS ONE 2014; 9(1): e86405. 24489723 10.1371/journal.pone.0086405 PMC3904875

[23] Lopez-de-Andres A Jiménez-García R Aragón-Sánchez J National trends in incidence and outcomes in lower extremity amputations in people with and without diabetes in Spain, 2001–2012. Diabetes Res Clin Pract 2015; 108(3): 499–507. 25866357 10.1016/j.diabres.2015.01.010

[24] Ikonen TS Sund R Venermo M Fewer major amputations among individuals with diabetes in Finland in 1997–2007: a population-based study. Diabetes Care 2010; 33(12): 2598–2603. 20807872 10.2337/dc10-0462 PMC2992197

[25] Jude EB Oyibo SO Chalmers N Peripheral arterial disease in diabetic and nondiabetic patients: a comparison of severity and outcome. Diabetes Care 2001; 24(8): 1433–1437. 11473082 10.2337/diacare.24.8.1433

[26] Kurichi JE Kwong PL Reker DM Clinical factors associated with prescription of a prosthetic limb in elderly veterans. J Am Geriatr Soc 2007; 55(6): 900–906. 17537091 10.1111/j.1532-5415.2007.01187.x PMC3700729

[27] Davies B Datta D. Mobility outcome following unilateral lower limb amputation. Prosthet Orthot Int 2003; 27(3): 186–190. 14727699 10.1080/03093640308726681

[28] Heikkinen M Saarinen J Suominen V Lower limb amputations: differences between the genders and long-term survival. Prosthet Orthot Int 2007; 31(3): 277–286. 17979013 10.1080/03093640601040244

[29] Jeffcoate WJ Margolis DJ. Incidence of major amputation for diabetes in Scotland sets a target for us all. Diabetes care 2012; 35(12): 2419–2420. 23173127 10.2337/dc12-1143 PMC3507597

[30] Jonasson JM Ye W Sparén P Risks of nontraumatic lower-extremity amputations in patients with type 1 diabetes: a population-based cohort study in Sweden. Diabetes Care 2008; 31(8): 1536–1540. 18443192 10.2337/dc08-0344 PMC2494662

[31] Peters SE Huxley R Woodward M. Diabetes as risk factor for incident coronary heart disease in women compared with men: a systematic review and meta-analysis of 64 cohorts including 858,507 individuals and 28,203 coronary events. Diabetologia 2014; 57(8): 1542–1551. 24859435 10.1007/s00125-014-3260-6

[32] Peters SAE Huxley RR Woodward M. Diabetes as a risk factor for stroke in women compared with men: a systematic review and meta-analysis of 64 cohorts, including 775,385 individuals and 12,539 strokes. Lancet 2014; 383(9933): 1973–1980. 24613026 10.1016/S0140-6736(14)60040-4

[33] Beekman CE Axtell LA. Prosthetic use in elderly patients with dysvascular above-knee and through-knee amputations. Phys Ther 1987; 67(10): 1510–1516. 3659135 10.1093/ptj/67.10.1510

[34] Tentolouris N Al-Sabbagh S Walker MG Mortality in diabetic and nondiabetic patients after amputations performed from 1990 to 1995: a 5-year follow-up study. Diabetes care 2004; 27(7): 1598–1604. 15220234 10.2337/diacare.27.7.1598

[35] Sandnes DK Sobel M Flum DR. Survival after lower-extremity amputation1,2. J Am Coll Surgeons 2004; 199(3): 394–402. 10.1016/j.jamcollsurg.2004.05.270 15325609

